# Hydroxychloroquine in COVID-19 Patients: Pros and Cons

**DOI:** 10.3389/fphar.2020.597985

**Published:** 2020-11-19

**Authors:** Nour K. Younis, Rana O. Zareef, Sally N. Al Hassan, Fadi Bitar, Ali H. Eid, Mariam Arabi

**Affiliations:** ^1^ Faculty of Medicine, American University of Beirut Medical Center, Beirut, Lebanon; ^2^ Pediatric Department, Division of Pediatric Cardiology, American University of Beirut Medical Center, Beirut, Lebanon; ^3^ Department of Basic Medical Sciences, College of Medicine, QU Health, Qatar University, Doha, Qatar; ^4^ Biomedical and Pharmaceutical Research Unit, QU Health, Qatar University, Doha, Qatar; ^5^ Department of Pharmacology and Toxicology, Faculty of Medicine, American University of Beirut, Beirut, Lebanon

**Keywords:** SARS-COV-2, COVID-19, hydroxychloroquine, chloroquine, drug discovery, drug repurposing

## Abstract

The pandemic of COVID-19, caused by SARS-CoV-2, has recently overwhelmed medical centers and paralyzed economies. The unparalleled public distress caused by this pandemic mandated an urgent quest for an effective approach to manage or treat this disease. Due to their well-established anti-infectious and anti-inflammatory properties, quinine derivatives have been sought as potential therapies for COVID-19. Indeed, these molecules were originally employed in the treatment and prophylaxis of malaria, and later in the management of various autoimmune rheumatic and dermatologic diseases. Initially, some promising results for the use of hydroxychloroquine (HCQ) in treating COVID-19 patients were reported by a few *in vitro* and *in vivo* studies. However, current evidence is not yet sufficiently solid to warrant its use as a therapy for this disease. Additionally, the therapeutic effects of HCQ are not without many side effects, which range from mild gastrointestinal effects to life-threatening cardiovascular and neurological effects. In this review, we explore the controversy associated with the repurposing of HCQ to manage or treat COVID-19, and we discuss the cellular and molecular mechanisms of action of HCQ.

## Introduction

Coronaviruses (CoVs) belong to the Coronaviridae family, and usually cause mild acute respiratory illnesses or “common cold” (Su et al., 2016). In December 2019, pneumonia cases of unknown etiology were reported in China (Huang et al., 2020). Within a few weeks, the cause of these cases appeared to be a novel coronavirus (CoV). This novel virus shares around 96% with bat-CoV RaTG13 and around 80% sequence similarity with the SARS-CoV (Lu et al., 2020; Zhou et al., 2020b). Hence, it was given the name SARS-CoV-2, and the disease it causes was called coronavirus infectious disease 2019, or shortly COVID-19.

COVID-19 has imparted serious threat to the global economy and health system. At the time of writing this manuscript (August 11, 2020), over 20 million cases and more than 700,000 deaths related to COVID-19 infection were reported globally (WHO, 2020a). Research efforts linked the origin of SARS-CoV-2 to bat-to-human transmission through an unidentified intermediate host. Human-to-human transmission can then take place through respiratory droplets (Salata et al., 2019).

SARS-CoV-2 infection may induce a wide spectrum of illnesses, with patient conditions range from being asymptomatic to severely ill. Indeed, various clinical symptoms with multi-organ involvement related to COVID-19 infection have been reported (Guan et al., 2020). These include respiratory, gastrointestinal, renal, neurologic and integumentary manifestations (Adhikari et al., 2020; Recalcati, 2020). Some of these are severe and life-threatening such as acute respiratory distress syndrome, acute kidney failure, stroke, arrythmias and heart failure (Adhikari et al., 2020; Bangalore et al., 2020; Cheng J. et al., 2020; Guan et al., 2020; Liu et al., 2020; Recalcati, 2020).

Treatment of COVID-19 infection is mainly symptomatic and is highly dependent on the severity of the disease. It includes hydration, pain control, fever treatment, oxygen supplementation, and invasive mechanical ventilation if needed (Cascella et al., 2020). As COVID-19 continues to be a source of global morbidity and mortality, urgent need of effective antiviral drug against COVID-19 appears. While numerous laboratories and clinical studies focused their efforts toward developing therapeutic and prophylactic interventions, repurposing an already known drug for use as an antiviral drug may be the fastest and least expensive. Indeed, recently, the anti-malarial agent hydroxychloroquine (HCQ) has gained attention as a potential drug that can be repositioned for the management of COVID-19. Below, we discuss the therapeutic value of this drug, along with its adverse effects.

## Pharmacology of Chloroquine and Hydroxychloroquine

Chloroquine (CQ) and HCQ are produced and administered orally in tablet form (Pastick et al., 2020). CQ tablet consists of 500 mg of CQ phosphate. HCQ tablet is composed of 200 mg of HCQ sulfate (Pastick et al., 2020). The required dosage varies according to the treated disease (Sanofi-Aventis, 2019). For malarial prophylaxis, a weekly dosage of 6.5 mg/kg is prescribed to adult and pediatric patients (Sanofi-Aventis, 2019). However, a single dose should not exceed 400 mg. Patients are instructed to take two doses before travel to endemic countries and to continue the same dose until 1 month after return. A higher dosage of 2000 mg is used to treat acute malaria. On the contrary, a daily dosage of 200–600 mg is used to treat rheumatoid arthritis and systemic lupus erythematous (SLE) (Sanofi-Aventis, 2019).

For the treatment of COVID-19, the used daily dosages of HCQ have ranged between 800 and 1,600 mg. However, in one study, they defined the effective and safe dose of HCQ based on data reported by *in vitro* studies and clinical trials (Garcia-Cremades et al., 2020). They examined the relationship between viral load reduction and the dosing of HCQ in treated COVID-19 patients. In this study, it was concluded that a daily dose of HCQ should not exceed 800 mg (Garcia-Cremades et al., 2020). Higher dosages may lead to quicker reduction in viral load and clinical improvement. However, they may induce undesired side effects such as QT interval prolongation. For the therapy duration, the above-mentioned regimen should be given over 7 days (Garcia-Cremades et al., 2020).

HCQ and CQ bioavailability is around 70–80% (Furst, 1996). This makes their use in oral formulation appropriate for treating serious multi-organ diseases. Moreover, they are both recognized by their slowed clearance. CQ is cleared at a rate of 0.35–1 L/h/kg and HCQ is cleared at a rate of 96 ml/min (Ducharme and Farinotti, 1996; Furst, 1996). Their elimination half-lives are estimated at 40–50 days (Furst, 1996). CQ and HCQ are likely known to have large plasma volume of distributions of up to around 65,000 L and 44,257 L respectively (Browning, 2014). Given these pharmacokinetic properties of HCQ and CQ, the clinical course of patients treated with these medications might not be easily predicted particularly in patients with comorbid renal and liver diseases. In fact, these patients are prone to develop serious side effects owed to defective clearance and metabolism of CQ and HCQ. Similarly, CQ and HCQ may exert varying therapeutic effects in distinct patients depending on their renal and hepatic functions.

## Mechanism of Action

Quinine along with its derivatives CQ and HCQ are weak bases that belong to the 4-aminoquinolines family (Savarino et al., 2003; Manohar et al., 2014). Both CQ and HCQ have common targets and similar mechanisms of action. Numerous mechanisms of action contribute to the role of these two drugs in a specific or a group of diseases (Yao et al., 2020). Below we discuss the mechanisms of action of CQ and HCQ which are classified into two groups based on their ultimate results: anti-inflammatory and anti-infectious.

### Anti-infectious Activity of Chloroquine and Hydroxychloroquine

HCQ and CQ express anti-viral activity through interfering in various steps of the viral replication. One postulated mechanism is through impairing viral interaction with the target cell receptor by CQ thus hindering viral entry to the cell. This is accomplished by inhibiting the enzyme quinone oxidoreductase 2 (QR2) which is found in red blood cells (Kwiek et al., 2004). QR2 is vital for sialic acid biosynthesis which is a component of ligand recognition (Varki, 1997). Recent studies suggest that CQ and HCQ act by binding to both the sialic acids and the gangliosides, both of which are essential for SARS-CoV-2 entry to the host cell (Fantini et al., 2020). Besides, CQ alters viral and cellular protein glycosylation thus limiting viral-receptor interaction. This is thought to be the key mechanism by which CQ alters the interaction of SARS-CoV with the ACE2 receptor (Vincent et al., 2005). Furthermore, CQ interferes with the p38 mitogen-activated protein kinase (MAPK) pathway which is used by viruses for completion of viral replication cycle (Seitz et al., 2003; Wehbe et al., 2020).

Indeed, HCQ and CQ preferentially confine to acidic organelles (Manohar et al., 2014), and alkalinize the acidic vesicles needed for multiplication of some infectious agents. This effect was observed with multiple organisms including Tropheryma whipplei, *Coxiella* burnetii and others that need acidic environment for multiplication (Rolain et al., 2007). This increase in pH also impairs the function of several cellular enzymes affecting post-translational modification and limiting iron availability inside the cell (Manohar et al., 2014). Such a mechanism is used against retrovirus infection, where inhibition of post-translational glycosylation of the viral glycoprotein abrogates its interaction with the virus (Savarino et al., 2004). Correspondingly, proteolytic enzymes needed for viral protein processing are not activated in the presence of alkaline environment (Randolph et al., 1990). Similarly, by increasing lysosomal pH, CQ impairs endosome-dependent viral entry to the cell (Gay et al., 2012). This alkalinizing property was also found to constrain the uncoating process of some viral particles (Manohar et al., 2014). In addition, it appears that CQ boosts cytotoxic T lymphocyte response against viral infection through enhancing viral antigen presentation by dendritic cells (Accapezzato et al., 2005).

### Anti-inflammatory Activity of Chloroquine and Hydroxychloroquine

The anti-inflammatory effects of CQ and HCQ are owed to their ability to modulate immune mechanisms. Indeed, CQ/HCQ elicit their effects by virtue of their ability to weaken the immune response. For instance, HCQ suppresses the release of several pro-inflammatory cytokines. Indeed, it abrogates the production of IL-6 in both monocytes and T-lymphocytes, and the production of IL-1 alpha in monocytes alone (Sperber et al., 1993). CQ also prevents the production of interleukin beta and Tumor Necrosis Factor-alpha (TNF-α) from macrophages (Jeong and Jue, 1997; Bondeson and Sundler, 1998). Interestingly, CQ represses TNF-α function by several mechanisms including decreased translation of its message (Bondeson and Sundler, 1998), post-translational change to soluble form (Jeong and Jue, 1997) or regulating the receptor expression (Jeong et al., 2002). CQ can also inhibit the activity of phospholipases A1 and A2 (Manku and Horrobin, 1976; Löffler et al., 1985). CQ negatively affects protein catabolism and antigen presentation while sparing phagocytic ability in macrophages (Ziegler and Unanue, 1982). HCQ also targets lymphocytes function by suppressing T-cell activation via inhibiting calcium signaling (Goldman et al., 2000). Besides, by virtue of their ability to abrogate toll-like receptor signaling, CQ and HCQ provide crucial immunosuppressive effect that is needed in the treatment of autoimmune diseases (Kyburz et al., 2006). To note, the immunomodulatory effects induced by CQ and HCQ are inferred from their therapeutic uses in rheumatic diseases such as rheumatoid arthritis and SLE.

**FIGURE 1:**
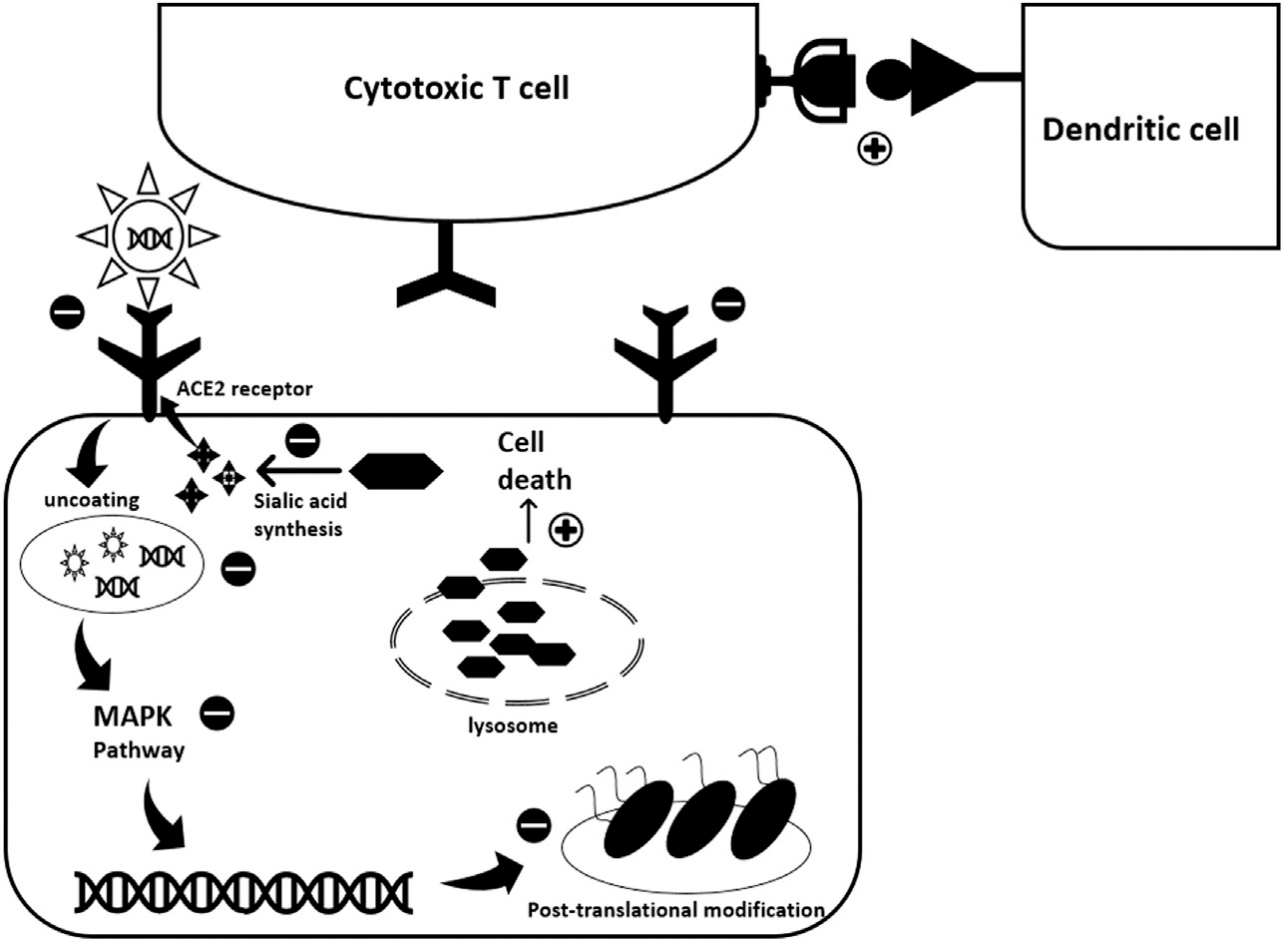
Potential antiviral activity of Hydroxychloroquine against SARS-CoV-2. HCQ exhibits its antiviral activity through interfering with various steps of the viral replication cycle. While exact mode of action against COVID-19 is not totally revealed, experience with previous viral infections highlights possible scenarios. First, HCQ acts at the pre-entry level. It inhibits SARS-CoV-2 entry into the host cell through three different mechanisms: 1) It binds to the sialic acids and gangliosides, key components used by the spike protein for viral entry; 2) It binds to spike protein-ACE2 receptor complex; 3) It inhibits the activity of Quinone oxidoreductase 2 (QR2) which is essential for sialic acid biosynthesis. Sialic acid is important for ligand recognition. Second, once the virus enters the cell HCQ inhibits the pH-dependent uncoating process by alkalinizing the acidic endosomes. This constrains viral-endosome fusion and consequent viral DNA release into the cytoplasm. The pH-dependent viral entry to the host cell was encountered with previous coronaviruses. Besides, HCQ impedes viral replication by blocking the p38 MAPK cascade. Also, it acts at the viral protein level where it interfere with post-translational modifications such as protein glycosylation. This alters SARS-CoV-2 proteins, affecting viral ability to interact with future host cells. The effect of HCQ extends to induce infected cell death through increasing lysosomal membrane permeability and consequently allowing proteolytic enzymes leakage into the cytoplasm. Finally, HCQ promotes the immune response through enhancing antigen expression by the dendritic cells thus activating cytotoxic T-lymphocytes.

The multiple cellular targets and effects of HCQ make it effective against many diseases. Despite some promising outcomes when used with COVID-19 patients, a clear mechanism of action in this particular disease has not yet been elucidated. However, based on the above-mentioned targets of HCQ in viral and autoimmune diseases, some potential cellular effects can be described (see Figure 1).

## Clinical Uses of Hydroxychloroquine

The medical use of quinine dates back to 1630 A.D. when the quinine powder, extracted from the tree of Cinchona was employed in the treatment of malaria (Schrezenmeier and Dörner, 2020). This was around 300 years before the medication and its derivatives, CQ and HCQ, were approved by the U.S. Food and Drug Administration (FDA), not only as treatment and prophylaxis for malaria, but also as treatment for rheumatic diseases (Table 1) (Schrezenmeier and Dörner, 2020). Currently, quinine derivatives are considered safe and well-tolerated medicines that are effective in treating a wide range of chronic autoimmune and rheumatic diseases such as anti-phospholipid syndrome, discoid or systemic lupus erythematous, Sjögren disease, juvenile idiopathic arthritis, psoriatic arthritis and rheumatoid arthritis, among others (Rynes, 1997; Lee et al., 2011; Al-Bari, 2015; Schrezenmeier and Dörner, 2020). CQ and HCQ are similarly effective in treating skin diseases such as dermatomyositis, cutaneous sarcoidosis, eosinophilic fasciitis, lichen planus and porphyria cutanea tarda (Rynes, 1997; Al-Bari, 2015). In the latter cases, they are used mainly when conventional therapies are contraindicated or ineffective (Rynes, 1997; Al-Bari, 2015).

**TABLE 1:** Key events portraying the well-approved clinical uses of hydroxychloroquine (HCQ) and chloroquine (CQ) along with the historical evolution of their utilization in the medical field (Schrezenmeier and Dörner, 2020).

Year	Event
1630	Quinine powder extracted from the tree of cinchona was used in the treatment of malaria
1934	Synthetic CQ was first produced
1949	The U.S Food and Drug Administration authorized the use of CQ in the management of rheumatic and skin diseases
1950	Synthetic HCQ was first produced
1955	The U.S Food and Drug Administration authorized the use of HCQ in the management of rheumatic and skin diseases

A multitude of distinctive immunomodulatory and anti-inflammatory properties made HCQ a clinically attractive drug (Al-Bari, 2015; Schrezenmeier and Dörner, 2020). Nevertheless, additional distinguishing effects have also been reported in the literature. They include anti-thrombotic, anti-neoplastic, and anti-microbial effects (Al-Bari, 2015; Schrezenmeier and Dörner, 2020). In addition, the use of HCQ in systemic lupus erythematous and rheumatoid arthritis patients has been associated with diminished rates of cardiovascular morbidities and diabetes mellitus, shedding the light on added favorable properties that need to be further investigated (Al-Bari, 2015; Schrezenmeier and Dörner, 2020). Similarly, HCQ was associated with improved glycemic and lipid profiles in these patients, and thus in improved overall survival and life quality (Al-Bari, 2015; Schrezenmeier and Dörner, 2020).

Other uses for HCQ have also been reported. For instance, when co-administered with doxycycline, HCQ can be effective in treating Q fever endocarditis (Raoult et al., 1990; Raoult et al., 1999). This regimen results in quicker recovery rates and infrequent relapses when compared to the originally adopted regimen (Raoult et al., 1999). Similarly, HCQ appears to be adequate for the management of Whipple disease and *Tropheryma whipplei* endocarditis (Boulos et al., 2004; Fenollar et al., 2013; Lagier et al., 2014). Moreover, HCQ is effective against a multitude of other microbial agents such as giardia, Ebola virus, hepatitis C, HIV and chikungunya (Al-Bari, 2015).

### Hydroxychloroquine and COVID-19

Recently, the emergence of the COVID-19 pandemic prompted an increased quest for potential therapies that could prove effective in controlling or improving the outcomes of the disease. Owing to its anti-viral properties, especially ones which showed its efficacy in diminishing actions of SARS-CoV-1 (Keyaerts et al., 2004; Colson et al., 2020; Hashem et al., 2020), HCQ was thought of being repurposed for fighting SARS-CoV-2 and the consequent COVID-19.

Several *in vitro* studies were conducted to assess the anti-SARS-CoV-2 properties of CQ and HCQ (Hashem et al., 2020; Liu et al., 2020; Wang et al., 2020;Yao et al., 2020). Importantly, both drugs appear to significantly inhibit SARS-CoV-2 replication (Hashem et al., 2020; Liu et al., 2020; Wang et al., 2020;Yao et al., 2020). Additionally, combined HCQ and azithromycin treatment caused a synergistic anti-SARS-CoV-2 effect *in vitro* (Andreani et al., 2020). Here, we discuss the controversy associated with the use of HCQ in COVID-19 patients by exploring supportive and opposing evidence.

### Pros

There are many advantages that make HCQ an attractive candidate. Not only it is safe, but it is also an effective medication with a broad spectrum of action covering various microbial and autoimmune diseases, likely by virtue of its ability to modulate the immune system (Wallace et al., 2012; Schrezenmeier and Dörner, 2020). Additionally, HCQ is a cheap medication with a good safety profile that has been garnered over hundreds of years of its use (Rynes, 1997; Savarino et al., 2003; Lee et al., 2011; Al-Bari, 2015; Schrezenmeier and Dörner, 2020; Zhou et al., 2020a). Importantly, it can be safely used in pregnant women as well (Costedoat-Chalumeau et al., 2003; Sperber et al., 2009).

The undesired side effects of this drug are also mild. They include gastrointestinal symptoms (nausea, vomiting and abdominal pain), along with cutaneous manifestations, and CNS symptoms (headache, dizziness, tinnitus and sleep disturbances) that are less frequently encountered (Rynes, 1997; Lee et al., 2011; Al-Bari, 2015; Littlejohn, 2020; Schrezenmeier and Dörner, 2020). Retinopathy is considered the most feared side effect of HCQ; yet, it is a rare manifestation that occurs mainly with prolonged use of high dosage therapy (Rynes, 1997; Wolfe and Marmor, 2010; Lee et al., 2011; Al-Bari, 2015; Littlejohn, 2020; Schrezenmeier and Dörner, 2020). Nevertheless, long-term monitoring and surveillance, and tight dosage regulation are associated with a reduced incidence of HCQ-induced retinopathy (Abdulaziz et al., 2018; Jorge et al., 2018). Other rare side effects, discussed in the following section, are encountered primarily in the presence of comorbid cardiovascular, renal and liver diseases (Gevers et al., 2020). Furthermore, HCQ is found to be less toxic and better tolerated than CQ (Rynes, 1997; Avina-Zubieta et al., 1998; Al-Bari, 2015; Liu et al., 2020; Schrezenmeier and Dörner, 2020; Zhou et al., 2020a).

Owed to its accessibility, effectiveness, and tolerability, HCQ has gained increased attention. It has been heavily examined in numerous studies as a potential treatment for the emerging pandemic of COVID-19. In this context, several studies, performed in different parts of the world, have discussed the benefits induced by the addition of HCQ to the conservative symptomatic therapies such as fluids, antipyretics and oxygen therapy (Table 2) (Gao et al., 2020; Gautret et al., 2020a; Gautret et al., 2020b; Million et al., 2020; Chen Z. et al., 2020).

**TABLE 2:** Clinical trials supporting HCQ use. The following databases were searched: Cochrane, embase, Medline, New England Journal of Medicine and PubMed. A total of five trials supported the use of HCQ in patients with COVID-19. All of them except one were observational cohort studies.

Evidence supporting the use of HCQ					
Study	Study type	Country	Population size	Results	Ref
Efficacy of hydroxychloroquine in patients with COVID-19: Results of a randomized clinical trial	Single-center RCT	China	62	A quicker recovery was noted in the HCQ-treated group compared to the control group. Studied outcomes included:• Time to clinical recovery • clinical progression • radiological progression	(Chen Z. et al., 2020)
Hydroxychloroquine and azithromycin as a treatment of COVID-19: Results of an open-label non-randomized clinical trial	Single-center, single-arm, non-randomized clinical trial	France	36	A quicker reduction in viral load was noted in the HCQ-treated group. A synergistic effect was prompted by the addition of azithromycin to the HCQ regimen. Studied outcomes included: • Virologic clearance achieved after 6 days of treatment • time to negative conversion • clinical progression • experienced side effects	(Gautret et al., 2020a)
Clinical and microbiological effect of a combination of hydroxychloroquine and azithromycin in 80 COVID-19 patients with at least a 6-day follow up: A pilot observational study	Single-center, uncontrolled, non-comparative observational study	France	80	Quicker reduction in viral load, shorter hospital stay, and improved outcomes were noted among the patients after an average of 5 days of treatment. Studied outcomes included: • Virologic clearance achieved up till day 12 of treatment • clinical outcomes • length of hospital stay	(Gautret et al., 2020b)
Early treatment of COVID-19 patients with hydroxychloroquine and azithromycin: A retrospective analysis of 1,061 cases in marseille, France	Single-center non-comparative, retrospective study	France	1,061	The use of HCQ combined with azithromycin was linked to improved mortality, clinical outcomes, and virologic clearance. Studied outcomes included: • Mortality clinical worsening (need for intensive care, and prolonged hospital stay) • virologic clearance	(Million et al., 2020)
Low dose of hydroxychloroquine reduces fatality of critically ill patients with COVID-19	Single-center comparative, retrospective study	China	550	The use of HCQ in critically ill COVID-19 patients was associated with improved survival, and reduced mortality rate. Studied outcomes included: • Length of hospital stay • mortality rate	(Yu, 2020)

Prior to the marked global propagation of the disease, trials using this drug has already started in China (Gao et al., 2020). In this context, it was reported that HCQ is more effective than conventional symptomatic treatment as per data derived from more than 100 patients (Gao et al., 2020). Indeed, treated patients had reduced disease severity, improved radiological findings, quicker virus clearance, and earlier recovery (Gao et al., 2020). Nonetheless, this study has several limitations. First, it is a non-randomized observational study with a limited number of participants. Second, the age and the pre-COVID-19 clinical status of the treated patients are not explicitly stated.

Similarly, *Zhaowei et al* assessed the efficacy of HCQ in a cohort of 62 SARS-CoV-2 positive patients (Chen Z. et al., 2020). Time to clinical recovery (TTCR), body temperature recovery time, and cough remission time were significantly reduced in the HCQ-treated group when compared to the control group (Chen Z. et al., 2020). Additionally, faster resolution of pneumonia was reported in 80.6% of the HCQ-treated patients vs. 54.8% of the control patients (Chen Z. et al., 2020). In this study, the diagnosis of COVID-19 was confirmed through several parameters including clinical, laboratory, physical and radiological findings. This minimizes the risk of missing a COVID-19 case and also the risk of misdiagnosing patients with COVID-19-like symptoms. The study is a randomized controlled clinical trial. However, the study is not blinded and is limited by the small number of participants. Similarly, patients with serious and critical COVID-19 were excluded as well as those with severe pre-existing medical conditions including arrhythmia, severe liver and renal diseases, and retinal diseases. This makes the selected cohort less susceptible to HCQ associated side effects.

A French trial included a total of 36 patients, 20 of whom received HCQ and 16 received control therapy (Gautret et al., 2020a). Azithromycin was added to the treatment of six HCQ-treated patients in order to avoid superimposed bacterial infection. These patients received daily echocardiographic monitoring (Gautret et al., 2020a). In this trial, HCQ was found to be superior to supportive therapy. After 7 days of treatment, the viral load was reduced in 70% of the HCQ-treated patients (Gautret et al., 2020a). Similarly, addition of azithromycin to HCQ resulted in quicker viral clearance when compared to HCQ alone. In fact, a synergistic reduction in the viral load was induced by this combination (Gautret et al., 2020a). In a second non-comparative study, *Gautret et al* revisited the benefit of HCQ incorporation in the management of COVID-19 patients, in a cohort of 80 patients (Gautret et al., 2020b). 81.3% of the patients had mild disease with favorable prognosis (Gautret et al., 2020b). 5% were asymptomatic and around 15% had moderate to severe disease requiring oxygen therapy. Three patients were admitted to the intensive care unit (Gautret et al., 2020b). A combination of azithromycin (500 mg on day 1, followed a course of 4 days of 250 mg daily) and HCQ (600 mg daily over 10 days) was given to all patients (Gautret et al., 2020b). After at least 3 days of treatment, 78 patients had improved clinical outcomes, early recovery, and reduced viral load. However, one patient died, and one remained in the intensive care unit despite treatment (Gautret et al., 2020b). The first study conducted by *Gautret el al* is a single-center non-randomized clinical trial limited by the lack of randomization and blinding and the minimal number of enrolled participants. It is likely limited by the lack of adequate follow up. Additionally, patients with retinopathy, QT prolongation and G6PD deficiency, who are prone to develop HCQ life-threatening side effects, were excluded. This reduces the incidence of serious side effects among the treated patients. Similarly, the second study is a non-randomized non-comparative observational study that is likely limited by the small number of participants and the imposed exclusion criteria. In fact, the evidence derived from these studies is not considered of high-quality owed to the limited sample size and the enhanced risk of selection bias.

Other trials examined the effectiveness of the same combination in a cohort of 1,061 confirmed inpatients (Million et al., 2020). In this study, the effect of this combination on mortality, recovery and viral shedding was determined. Findings showed that the virus cleared in 91.7% of the patients after less than 10 days of treatment. 4.4% of the patients, with a higher original viral load required a longer period of 10 days to clear the virus (Million et al., 2020). Unfavorable clinical outcome was noted in 46 patients (4.3%). Similarly, eight patients, accounting for 0.75%, died due to respiratory failure. Cardiac toxicity was not reported in any of the patients (Million et al., 2020). This study demonstrated that HCQ and azithromycin can be safely used in patients with early disease, particularly in the absence of associated complications. They accelerate recovery and improve overall clinical outcomes (Million et al., 2020). Unlike the previously mentioned studies, this study has a larger cohort of participants. Plasma levels of medications were monitored adequately in most patients and the diagnosis of COVID-19 was based on sufficient clinical and laboratory evidence. However, just like all retrospective studies, the study is subjected to the inherent limitations of retrospective studies denoted by the lack of control and randomization and the biased selection of the participants. Additionally, patients susceptible to HCQ toxicities were likely excluded from the studied cohort.

The above-mentioned trials have triggered a call for further investigations. In fact, out of 688 and 2122 COVID-19 related ongoing trials registered in the Chinese clinical trial registry and the U.S. National Library of Medicine respectively, 11 and 218 trials aim to examine the effectiveness of HCQ in COVID-19 patients (Chinese Clinical Trial Registry, 2020; U.S. National Library of Medicine, 2020a; U.S. National Library of Medicine, 2020b; U.S. National Library of Medicine, 2020c; U.S. National Library of Medicine, 2020d). Ultimately, it is hoped that these trials will provide a clearer understanding of the therapeutic role of HCQ in curing SARS-CoV-2 infection, and also in averting its propagation.

### Cons

#### Hydroxychloroquine and Viral Clearance in COVID Patients

Although HCQ is relatively safe to use in treating malaria and autoimmune diseases, COVID-19 patients may be more susceptible to its adverse reactions, in part because of the compromised function of vital organs secondary to SARS-CoV-2 infection (Gevers et al., 2020). Since HCQ is cleared by the kidney and the liver, severely ill patients, particularly ones with impaired renal or hepatic functions, are at increased risk of experiencing serious adverse reactions. Besides, drug-drug interactions are major causes of some of HCQ’s adverse events (Gevers et al., 2020).

HCQ has an estimated half-life of around 2 months, and is inadequately distributed in adipose tissues. Thus, monitoring for side effects, over a long period of time, is highly advised particularly in the presence of severe comorbid conditions (Gevers et al., 2020). Furthermore, HCQ’s adverse effects may mask or interfere with symptoms of specific illnesses such as COVID-19. This is especially important when evaluating their cardiovascular, neuropsychiatric and gastrointestinal side effects (Gevers et al., 2020).

Following the decision of The Health Ministry of France of permitting the use of HCQ to treat COVID-19, a prospective study assessed the outcomes of 11 patients that were hospitalized at Saint-Louis Hospital (Molina et al., 2020). Those patients received 600 mg/d of HCQ in combination with azithromycin for 10 days (500 mg day 1 and 250 mg days 2–5) (Molina et al., 2020). Among them, eight had underlying comorbid diseases (Molina et al., 2020). As the treatment started, 10 patients were febrile and on oxygen therapy. After 5 days of treatment, one patient passed away and two were moved to the intensive care unit. Furthermore, in one patient, the treatment was terminated after 4 days due to QT prolongation (Molina et al., 2020). After five to 6 days of treatment initiation, the nasopharyngeal swabs of eight patients were still positive for SARS-CoV-2 (Molina et al., 2020). However, these results contradicted the optimistic outcomes provided by an earlier study where 70% of patients treated with HCQ had negative PCR testing by day 6, compared to only 12.5% of patients in the control group (Gautret et al., 2020a). Congruently, no significant difference in the rate of viral clearance, hospital stay, radiologic findings or temperature regulation, between control and HCQ treated groups was also reported (Chen J. et al., 2020). Nevertheless, these two studies conducted by *Molina el al* and *Chen et al* are both limited by the small number of enrolled patients. The first study is likely subjected to several limitations owed to the lack of control and randomization and the inherent errors associated with observational studies.

The RECOVERY trial is a large multi-center randomized controlled trial that compares several treatments to standard management in patients with COVID-19 (Horby et al., 2020). Preliminary results depicting the difference between HCQ and standard care have shown no improvement in the clinical outcomes of the HCQ-treated group (Horby et al., 2020). HCQ was not linked with improved mortality in the treated group. Yet, it imposed an increase in the duration of hospitalization and an enhanced risk of deterioration and progression to assisted respiration (Horby et al., 2020). The SOLIDARITY trial is another multi-national multi-center randomized controlled clinical trial issued by WHO (WHO, 2020b). It compares several proposed anti-COVID-19 therapies to usual care. Owed to absence of or minimal benefit induced by HCQ, the committee has decided to discontinue the use of HCQ in this trial (WHO, 2020b). Furthermore, the ORCHID Study, a third multi-center placebo-controlled randomized clinical trial comparing HCQ to standard therapy, was also terminated by the National Institutes of Health due to the lack of benefit produced by HCQ (NIH, 2020).

Moreover, a recently published multicenter randomized clinical trial performed in Brazil has compared the efficacy of standard care alone to each of standard care plus HCQ and standard care plus combined HCQ and azithromycin in hospitalized patients with mild to moderate disease (Cavalcanti et al., 2020). 667 patients were randomly assigned to one of the three groups. HCQ was given at a dosage of 800 mg/day divided into two doses for 7 days. Azithromycin was given at a dosage of 500 mg once daily for 7 days. No significant difference in clinical status was noted among the three treated groups at 15 days of treatment initiation (Cavalcanti et al., 2020). Additionally, more side effects were encountered by the HCQ and the HCQ plus azithromycin treated groups as compared to the control group. Prolonged QT interval and hepatic injury were among the witnessed side effects in this study (Cavalcanti et al., 2020). This study was subjected to multiple limitations. First, the number of assessed outcomes was limited. Hence, the role of each of HCQ and azithromycin in treating COVID-19 cannot be objectively assessed and based on this study since unstudied benefits induced by HCQ and azithromycin may be easily missed. Second, no blinding was applied in this study. Finally, adherence to treatment regimen cannot be asserted owed to the increased demand for and the lack of these medications in some of the enrolled hospitals. This may result in biased and inconsistent outcomes.

Besides, in a recently published study, SARS-CoV-2 infection was found to be resistant to CQ in lung cells positive for TMPRSS2, a cellular protease that facilitates the invasion of the cells by SARS-CoV-2 (Markus Hoffmann, 2020). The potential inhibitory effect induced by the expression of TMPRSS2 was not seen in non-pulmonary cell lines (Markus Hoffmann, 2020). This means that CQ, and likely HCQ, may not be effective in clearing SARS-CoV-2 infection in pulmonary tissues, and that initial *in-vitro* results supporting the use of HCQ in COVID-19 patients might have stemmed from experiments performed on non-pulmonary tissues (Markus Hoffmann, 2020). Congruently, use of HCQ for clearing SARS-CoV2 infection was not supported by preclinical evidence despite the different models employed such as mice or hamsters, or even *in vitro* studies (Funnell et al., 2020). This endorses the hypothesis suggested by Hoffman. In short, HCQ seems to be not suitable for treating human or human-like pulmonary tissues infected with SARS-CoV2 as concluded from these *in vitro* and *in vivo* studies.

As such, despite the positive outcome reported by some studies, whether HCQ is effective or not remains controversial. Furthermore, no solid evidence has validated the potent antiviral activity or clinical benefit of the combination of HCQ and azithromycin in curing hospitalized COVID-19 patients with moderate to severe disease. Table 3 highlights the unfavorable results reported by some of the completed clinical trials.

**TABLE 3:** Clinical trials against HCQ use. The following databases were searched: Cochrane, Embase, Medline, New England Journal of Medicine and PubMed. A total of four trials (two randomized controlled trials (RCT) and two cohort studies) showed no significant improvement in clinical outcome and mortality when comparing the HCQ-treated group to the control group.

Evidence against the use of HCQ					
Study	Study type	Country	Population size	Results	Ref
A pilot study of hydroxychloroquine in treatment of patients with common coronavirus disease-19 (COVID-19)	Single-center RCT	China	30	No significant prognostic difference was noted between the HCQ-treated group and the control group.Comparable adverse events were experienced by each group. Studied outcomes included: • Time to negative conversion • time for body temperature normalization • radiological progression• adverse events experienced by both groups	(Chen J. et al., 2020)
Hydroxychloroquine in patients with mainly mild to moderate coronavirus disease 2019: Open label, randomised controlled trial	Multi-center RCT	China	150	No significant prognostic difference was noted between the HCQ-treated group and the control group. More adverse events were observed in the treated group.Studied outcomes included: • Time to negative conversion • adverse events experienced by both groups	(Tang et al., 2020)
Association of treatment with hydroxychloroquine or azithromycin with in-hospital mortality in patients with COVID-19 in New York state	Multi-center retrospective cohort study	United States, New York	1,438	There was no significant improvement in mortality rate in hospitalized COVID-19 patients treated with HCQ, azithromycin or HCQ + azithromycin. Studied outcomes included: • In-hospital mortality • incidence of fatal cardiac events	(Rosenberg et al., 2020)
Observational study of hydroxychloroquine in hospitalized patients with Covid-19	Single-center, observational study	United States, New York	1,446	The use of HCQ had no significant effect on clinical outcomes and in-hospital mortality.Studied outcomes included: • Clinical worsening (denoted by the need for intubation) • death	(Geleris et al., 2020)

#### Serious Side Effects of Hydroxychloroquine

**FIGURE 2:**
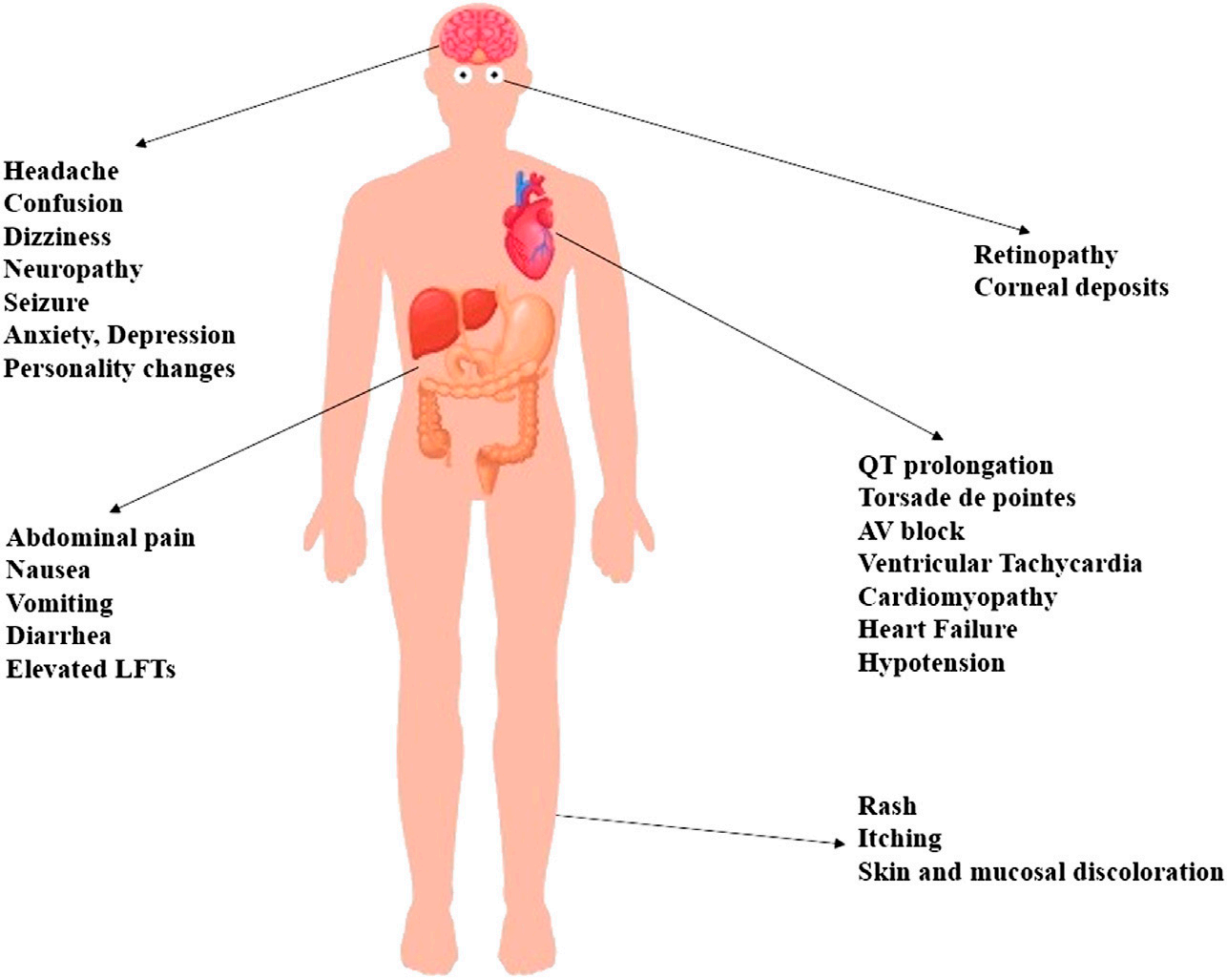
A multitude of side effects may be elicited by the use of HCQ. Most of the experienced side effects are mild and well-tolerated ones that include cutaneous, gastrointestinal and neurological symptoms denoted by itching, skin rashes, abdominal pain, nausea, vomiting, diarrhea, headache, and dizziness. Life-threatening cardiovascular side effects are particularly encountered in critically ill patients with comorbid hepatic or renal diseases. Similarly, the toxicity of HCQ can be potentiated by the coadministration of drugs that may affect the metabolism of HCQ, or enhance its pro-arrhythmic effects. Retinopathy constitutes another feared side effect associated with chronic use of high-dose HCQ. Furthermore, patients may experience serious gastrointestinal events such as liver injury, and neuropsychiatric events such as confusion, seizure, mood changes and psychosis.

In addition to its above-mentioned well-tolerated gastrointestinal and cutaneous adverse reactions, HCQ may lead to serious cardiotoxic, metabolic and neuropsychiatric manifestations, as depicted in Figure 2. Indeed, HCQ may lead to a multitude of cardiac events denoted by bundle branch block, complete AV block, QRS and QT prolongation, Torsades de pointes, and ventricular tachyarrhythmia (Jordan et al., 1999; Marquardt and Albertson, 2001; Chen et al., 2006; Kruisselbrink and Zaki Ahmed, 2010; Gevers et al., 2020). Similarly, patients on HCQ may develop cardiotoxicity secondary to HCQ-induced hypokalemia (Jordan et al., 1999; Marquardt and Albertson, 2001; Chen et al., 2006; Kruisselbrink and Zaki Ahmed, 2010). These patients may become prone to serious life-threatening hypotensive episodes especially in the setting of prolonged intake and overdose (Jordan et al., 1999; Marquardt and Albertson, 2001; Chen et al., 2006; Kruisselbrink and Zaki Ahmed, 2010).

Long-term use of HCQ has been associated, in rare cases, with advanced cardiomyopathy, as well as subsequent cardiovascular compromise and heart failure (Costedoat-Chalumeau et al., 2007; Hartmann et al., 2011; Muthukrishnan et al., 2011; Joyce et al., 2013; Zhao et al., 2018). Notably, HCQ cardiotoxicity is primarily encountered in patients with preexisting liver or kidney diseases, as well as in those taking medications that may affect HCQ metabolism or potentiate its side effects (Chen et al., 2006; Gevers et al., 2020). For instance, the risk of QT prolongation is greater when HCQ is added to other QT-prolonging drugs such as macrolides (Gevers et al., 2020). Besides, pediatric patients are also susceptible to HCQ’s pro-arrhythmic effects, even if only small doses are used (Erickson et al., 2020). Hence, precise dosing and careful monitoring are both required to avoid fatal cardiotoxicities of HCQ.

In a recent study, male gender, older age and concurrent intake of NSAIDs were identified as potential risk factors for HCQ cardiotoxicity (Cohen et al., 2020). In addition, it appears that CQ confers a higher risk of cardiotoxicity as compared to HCQ (Cohen et al., 2020). Contextually, HCQ cardiotoxicity becomes of utmost relevance in COVID-19 patients. Indeed, two separate studies show that cardiac involvement is a predictor of mortality in COVID-19 patients (Guo et al., 2020; Shi et al., 2020). This explains the higher incidence of complications and adverse events in the group of HCQ-treated COVID-19 patients, as evidenced by an open label randomized clinical trial of 150 Chinese patients (Tang et al., 2020).

Hypoglycemia is yet another serious side effect exerted, albeit rarely, by HCQ (Cansu and Korkmaz, 2008; Sheikhbahaie et al., 2016; Richard De-Heer, 2018). Indeed, evidence stemming from both *in vitro* and clinical studies underscores the role of HCQ in reducing blood glucose levels. This is primarily achieved through the potentiation of the hypoglycemic effects of insulin (Cynober et al., 1987). It appears that HCQ increases plasma levels of insulin via the downregulation of its intracellular breakdown as well as the enhancement of intracellular accumulation (Cynober et al., 1987). Additionally, HCQ elicits this hypoglycemic effect by reducing the rate of glucose receptor recycling, and also by promoting insulin-dependent cellular uptake of glucose (Cynober et al., 1987).

Other undesired outcomes such as psychiatric and neuromuscular adverse effects have also been associated with HCQ use, particularly with prolonged use of increased doses. Some of these adverse effects include anxiety, agitation, depression and personality changes (Manzo et al., 2017; Gevers et al., 2020). Moreover, confusion, headache, neuropathy, seizure, visual disturbances and weakness represent reversible CNS manifestations of high dose-use of HCQ (Estes et al., 1987; Stein et al., 2000; Kwon et al., 2010; Vinciguerra et al., 2015; Gevers et al., 2020).

Table 4 depicts side effects experienced by COVID-19 patients as well as the incidence of these adverse effects among the treated patients. Overall, the incidence of side effects has ranged between 0.06% and 33.67%. This depends largely on the administered dose of HCQ and the co-existence of cardiac, hepatic and renal diseases that might potentiate the toxicity of HCQ. Most side effects were mild cutaneous, gastrointestinal and neurologic. Nonetheless, serious life-threatening side effects such as torsades de points and QT interval prolongation were likely encountered by these patients.

**TABLE 4:** Side effects experienced by COVID-19 patients and their incidence among the treated patients.

Study	Side effects	Incidence of side effects	Ref
Efficacy of hydroxychloroquine in patients with COVID-19: Results of a randomized clinical trial	Skin rash (1) headache (1)	6.5% (2/31)	(Chen Z. et al., 2020)
			
Clinical and microbiological effect of a combination of hydroxychloroquine and azithromycin in 80 COVID-19 patients with at least a 6-day follow up: A pilot observational study	Blurry vision (1) nausea/Vomiting (2) diarrhea (4)	8.75% (7/80)	(Gautret et al., 2020b)
			
Early treatment of COVID-19 patients with hydroxychloroquine and azithromycin: A retrospective analysis of 1,061 cases in marseille, France	Abdominal pain (3), diarrhea (12), nausea (1), vomiting (1), headache (3), insomnia (2), blurry vision (2), skin rash (2)	2.35% (25/1,061)	(Million et al., 2020)
			
No evidence of rapid antiviral clearance or clinical benefit with the combination of hydroxychloroquine and azithromycin in patients with severe COVID-19 infection	QT interval prolongation (1)	9.1% (1/11)	(Molina et al., 2020)
			
A pilot study of hydroxychloroquine in treatment of patients with moderate COVID-19	Diarrhea (4) elevated liver enzymes (4)	26.67% (4/15)	(Chen J. et al., 2020)
			
Effect of hydroxychloroquine in hospitalized patients with COVID-19: Preliminary results from a multi-centre, randomized, controlled trial	Torsades de pointes (1)	0.06% (1/1,561)	(Horby et al., 2020)
			
Hydroxychloroquine with or without azithromycin in mild-to-moderate Covid-19	QT interval prolongation (13), arrhythmia (3), bradycardia (1), supraventricular tachycardia (2), pneumothorax (1), nausea (9), anemia (14), bloodstream infection (1), elevated liver enzymes (17), itching (1), hypoglycemia (1), elevated bilirubin level (5), thrombocytopenia (14), leukopenia (3), lymphopenia (17)	33.67% (67/199)	(Cavalcanti et al., 2020)

### Hydroxychloroquine as Pre- and Post-exposure Prophylaxis for COVID-19

To date, no medication has been approved for pre- and post-exposure prevention of COVID-19. Adequate quarantine and monitoring of clinical symptoms remain the mainstay of post-exposure prophylaxis. Similarly, appropriate practicing of social distancing and proper utilization of personal protection equipment, including face masks and googles, continue to be the core means of COVID-19 pre-exposure prevention. Nonetheless, the role of HCQ in preventing COVID-19 pre- and post-exposure has been tackled in various clinical studies. *Boulware et al* performed a double-blinded randomized clinical trial involving 821 American and Canadian individuals who were exposed to a confirmed case of COVID-19 at home or occupation (Boulware et al., 2020). The participants were divided into two groups based on the degree of personal protection at the time of exposure: 1) group of high-risk exposure and 2) group of moderate risk exposure. Exposed Individuals with and without a face mask were considered at moderate- and high-risk respectively (Boulware et al., 2020). Furthermore, participants were given randomly placebo or HCQ. A cumulative HCQ dose of 3,800 mg, divided over five days, was provided to the treated group. After 14 days of follow up, there was no significant difference in the number of newly diagnosed COVID-19 cases among the placebo and the treated groups. Additionally, treated patients were subjected to more side effects with most side effects being self-limited gastrointestinal and neurologic effects (Boulware et al., 2020).

Interestingly, this trial had several limitations including inadequate confirmation of exposure, and inappropriate diagnosis of COVID-19 based on clinical symptoms in the absence of molecular confirmation. Indeed, participants who developed clinical symptoms similar to those of COVID-19 were considered SARS-CoV2 positive. Their infection was not proven positive through SARS-CoV2 polymerase Chain Reaction (PCR) testing. Furthermore, the median age of the enrolled participants was 40 years and most were aged between 33 and 50 years. This means that most of the enrolled participants were healthy young individuals. As a result, the prophylactic effect of HCQ can be better assessed through larger randomized clinical trials that involve older patients with pre-existing comorbid conditions.

The pre-exposure prophylactic effect of HCQ has been likely investigated in a multitude of complete and ongoing studies. In one double-blinded randomized clinical trial, the efficacy of HCQ in preventing COVID-19 was examined among 1,483 American and Canadian healthcare workers who are significantly exposed to COVID-19 patients in high-risk areas such as emergency departments, COVID-19 units and intensive care units (Rajasingham et al., 2020). The participants were assigned to three groups: 1) HCQ group 1, provided with a dose of 400 mg once weekly for 12 weeks, 2) HCQ group 2, provided with a dose of 800 mg twice weekly for 12 weeks, and 3) placebo group (Rajasingham et al., 2020). After 12 weeks of follow up, no significant difference in the incidence of COVID-19 was detected among the three groups (Rajasingham et al., 2020). However, just like the previous study, this study was limited by the lack of adequate PCR testing, and also by the inherent error associated with the use of PCR in confirming COVID-19. Similarly, the diagnosis of COVID-19 in many participants was made based on clinical judgment and was not confirmed through laboratory testing.

## Conclusion

Evidence on the effectiveness and safety of HCQ in treating COVID-19 infection is still controversial. Most of the available studies are non-randomized with preliminary results. We argue that multi-center placebo-controlled randomized clinical trials are urgently needed to assess the efficacy, safety as well as determining the best dosing regimen of HCQ. It is also essential to assess longer-term effects, and thus a thorough examination of upcoming results reported by high-quality ongoing trials is much needed (see Table 5).

**TABLE 5:** | Ongoing clinical trials. Owed to the scarcity of reliable evidence, hundreds of clinical trials were initiated in many parts of the world. Here, we searched the databases of Cochrane, embase, Medline, New England Journal of Medicine and PubMed along with the clinical trial registry (ClinicalTrial.gov), and we selected randomly some ongoing trials.

Ongoing clinical trials					
Study	Study type	Country	Population size	Results	Ref
Azithromycin added to hydroxychloroquine for patients admitted to intensive care due to coronavirus disease 2019 (COVID-19)-protocol of randomised controlled trial AZIQUINE-ICU	Multi-center, double-blind, RCT	Czech republic	Not yet indicated	Outcomes to be studied: • Clinical worsening • mortality	(Duska et al., 2020)
			
Treatment with hydroxychloroquine vs hydroxychloroquine + nitazoxanide in COVID-19 patients with risk factors for poor prognosis: A structured summary of a study protocol for a randomised controlled trial	Single-center, single-blind, RCT	Mexico	86	Outcomes to be studied: • Need for mechanical ventilation • death	(Calderon et al., 2020)
			
Test and treat COVID 65 plus - hydroxychloroquine vs. placebo in early ambulatory diagnosis and treatment of older patients with COVID19: A structured summary of a study protocol for a randomised controlled trial	Multi-center, double-blind, RCT	Germany	300–400	Outcomes to be studied: • Need for hospitalization • death	(Gopel et al., 2020)
			
The COVIRL-001 trial: A multicentre, prospective, randomised trial comparing standard of care (SOC) alone, SOC plus hydroxychloroquine monotherapy or SOC plus a combination of hydroxychloroquine and azithromycin in the treatment of non- critical, SARS-cov-2 PCR-positive population not requiring immediate resuscitation or ventilation but who have evidence of clinical decline: A structured summary of a study protocol for a randomised controlled trial	RCT	Ireland	351	Outcomes to be studied: • Time to progression to intubation or non-invasive ventilation • need for high-dose corticosteroids • death	(Feeney et al., 2020)
			
Use of hydroxychloroquine in patients with COVID-19: A randomized controlled clinical trial	RCT	Saudi Arabia	200	Outcomes to be studied: • Time to viral clearance • mortality	(U.S. National Library of Medicine, 2020b)
			
Single-center, phase II, randomized double-blind, placebo-controlled study of hydroxychloroquine compared to placebo as treatment for severe acute respiratory syndrome coronavirus 2 (SARS-cov-2) infection	Single-center, double-blind, RCT	United States, New York	120	Outcomes to be studied: • Clinical improvement • need for mechanical ventilation	(U.S. National Library of Medicine, 2020d)
			
Hydroxychloroquine in SARS-cov-2 (COVID-19) pneumonia trial	Single-center RCT	United States, Washington	120	Outcomes to be studied: • Change from baseline oxygenation • length of ICU/hospital stay • need for oxygen therapy/mechanical ventilation • mortality • incidence of fatal cardiac events	(U.S. National Library of Medicine, 2020c)
			
Norwegian coronavirus disease 2019 (NO COVID-19) pragmatic open label study to assess early use of hydroxychloroquine sulfate in moderately severe hospitalised patients with coronavirus disease 2019: A structured summary of a study protocol for a randomised controlled trial	Single-center double-blind, RCT	Norway	202	Outcomes to be studied: • Rate of viral load reduction • need for intensive care • length of hospital stay • mortality • clinical status reached after 14 days of study initiation • laboratory profile	(Lyngbakken et al., 2020)

## Author Contributions

MA, FB and AE developed the idea and the review framework. NY, RZ, SA wrote the first draft of the article. AE did the final editing. All authors contributed to corrections and adjustment of subsequent iterations of the article. All authors approve and agree with the content.

## Conflict of Interest

The authors declare that the research was conducted in the absence of any commercial or financial relationships that could be construed as a potential conflict of interest.

## Abbreviations

**Abbreviations:** HCQ, Hydroxychloroquine; LFTs, Liver function tests; AV block, Atrioventricular block.

## References

[B1] Abdulaziz N. Shah A. R. Mccune W. J. (2018). Hydroxychloroquine. Curr. Opin. Rheumatol. 30, 249–255. 10.1097/bor.0000000000000500 29517495

[B2] Accapezzato D. Visco V. Francavilla V. Molette C. Donato T. Paroli M. (2005). Chloroquine enhances human CD8+ T cell responses against soluble antigens *in vivo* . J. Exp. Med. 202, 817–828. 10.1084/jem.20051106. 16157687 PMC2212941

[B3] Adhikari S. P. Meng S. Wu Y.-J. Mao Y.-P. Ye R.-X. Wang Q.-Z. (2020). Epidemiology, causes, clinical manifestation and diagnosis, prevention and control of coronavirus disease (COVID-19) during the early outbreak period: a scoping review. Infect. Dis. Poverty 9, 29 10.1186/s40249-020-00646-x. 32183901 PMC7079521

[B4] Al-Bari M. A. A. (2015). Chloroquine analogues in drug discovery: new directions of uses, mechanisms of actions and toxic manifestations from malaria to multifarious diseases. J. Antimicrob. Chemother. 70, 1608–1621. 10.1093/jac/dkv018. 25693996 PMC7537707

[B5] Andreani J. Le Bideau M. Duflot I. Jardot P. Rolland C. Boxberger M. (2020). *In vitro* testing of combined hydroxychloroquine and azithromycin on SARS-CoV-2 shows synergistic effect. Microb. Pathog. 145, 104228 10.1016/j.micpath.2020.104228. 32344177 PMC7182748

[B6] Avina-Zubieta J. A. Galindo-Rodriguez G. Newman S. Suarez-Almazor M. E. Russell A. S. (1998). Long term effectiveness of antimalarial drugs in rheumatic diseases. Ann. Rheum. Dis. 57, 582–587. 10.1136/ard.57.10.582. 9893568 PMC1752486

[B7] Bangalore S. Sharma A. Slotwiner A. Yatskar L. Harari R. Shah B. (2020). ST-segment elevation in patients with Covid-19—a case series. N. Engl. J. Med. 382, 2478–2480. 10.1056/NEJMc2009020 32302081 PMC7182015

[B8] Bondeson J. Sundler R. (1998). Antimalarial drugs inhibit phospholipase A2 activation and induction of interleukin lβ and tumor necrosis factor α in macrophages: implications for their mode of action in rheumatoid arthritis. Gen. Pharmacol. Vasc. Syst. 30, 357–366. 10.1016/s0306-3623(97)00269-3. 9510087

[B9] Boulos A. Rolain J.-M. Raoult D. (2004). Antibiotic susceptibility of Tropheryma whipplei in MRC5 cells. Antimicrob. Agents Chemother. 48, 747–752. 10.1128/aac.48.3.747-752.2004. 14982759 PMC353111

[B10] Boulware D. R. Pullen M. F. Bangdiwala A. S. Pastick K. A. Lofgren S. M. Okafor E. C. (2020). A randomized trial of hydroxychloroquine as postexposure prophylaxis for covid-19. N. Engl. J. Med. 383, 517–525. 10.1056/nejmoa2016638. 32492293 PMC7289276

[B11] Browning D. J. (2014). “Pharmacology of chloroquine and hydroxychloroquine.” in Hydroxychloroquine and chloroquine retinopathy. Editor Browning D. J. (New York, NY: Springer-Verlag), 35–63 *.*

[B12] Calderon J. M. Zeron H. M. Padmanabhan S. (2020). Treatment with Hydroxychloroquine vs Hydroxychloroquine + Nitazoxanide in COVID-19 patients with risk factors for poor prognosis: a structured summary of a study protocol for a randomised controlled trial. Trials 21, 504 10.1186/s13063-020-04448-2. 32513231 PMC7276957

[B13] Cansu D. U. Korkmaz C. (2008). Hypoglycaemia induced by hydroxychloroquine in a non-diabetic patient treated for RA. Rheumatology 47, 378–379. 10.1093/rheumatology/kem378. 18222983

[B14] Cascella M. Rajnik M. Cuomo A. Dulebohn S. C. Di Napoli R. (2020). Features, evaluation and treatment coronavirus (COVID-19). Statpearls [internet] Treasure Island (FL): StatPearls Publishing. 32150360

[B15] Cavalcanti A. B. Zampieri F. G. Rosa R. G. Azevedo L. C. P. Veiga V. C. Avezum A. (2020). Hydroxychloroquine with or without azithromycin in mild-to-moderate covid-19. N. Engl. J. Med. [Epub ahead of print] 10.1056/nejmoa2019014. PMC7397242 32706953

[B16] Chen C.-Y. Wang F.-L. Lin C.-C. (2006). Chronic hydroxychloroquine use associated with QT prolongation and refractory ventricular arrhythmia. Clin. Toxicol. 44, 173–175. 10.1080/15563650500514558. 16615675

[B17] Chen J. Liu D. Liu L. Liu P. Xu Q. Xia L. (2020). A pilot study of hydroxychloroquine in treatment of patients with common coronavirus disease-19 (COVID-19). J. Zhejiang Univ. 49, 215–219 [in Chinese, with English summary]. 10.3785/j.issn.1008-9292.2020.03.03. PMC8800713 32391667

[B18] Chen Z. Hu J. Zhang Z. Jiang S. Han S. Yan D. (2020). Efficacy of hydroxychloroquine in patients with COVID-19: results of a randomized clinical trial. medRxiv 10.1101/2020.03.22.20040758.

[B19] Cheng Y. Luo R. Wang K. Zhang M. Wang Z. Dong L. (2020). Kidney disease is associated with in-hospital death of patients with COVID-19. Kidney Int. 97, 829–838. 10.1016/j.kint.2020.03.005 32247631 PMC7110296

[B20] Chinese Clinical Trial Registry (2020). Trial Search. [Online] Available at: http://www.chictr.org.cn/searchprojen.aspx?title=Hydroxychloroquine&officialname=&subjectid=&secondaryid=&applier=&studyleader=&ethicalcommitteesanction=&sponsor=&studyailment=&studyailmentcode=&studytype=0&studystage=0&studydesign=0&minstudyexecutetime=&maxstudyexecutetime=&recruitmentstatus=0&gender=0&agreetosign=&secsponsor=&regno=&regstatus=0&country=&province=&city=&institution=&institutionlevel=&measure=&intercode=&sourceofspends=&createyear=0&isuploadrf=&whetherpublic=&btngo=btn&verifycode=&page=1 (Accessed July 16, 2020).

[B21] Cohen I. V. Makunts T. Moumedjian T. Issa M. Abagyan R. (2020). Determinants of cardiac adverse events of chloroquine and hydroxychloroquine in 20 years of drug safety surveillance reports. medRxiv 10.1101/2020.05.19.20107227. PMC7644696 33154498

[B22] Colson P. Rolain J.-M. Lagier J.-C. Brouqui P. Raoult D. (2020). Chloroquine and hydroxychloroquine as available weapons to fight COVID-19. Int. J. Antimicrob. Agents 55, 105932 10.1016/j.ijantimicag.2020.105932. 32145363 PMC7135139

[B23] Costedoat-Chalumeau N. Amoura Z. Duhaut P. Huong D. L. T. Sebbough D. Wechsler B. (2003). Safety of hydroxychloroquine in pregnant patients with connective tissue diseases: a study of one hundred thirty-three cases compared with a control group. Arthritis Rheum. 48, 3207–3211. 10.1002/art.11304. 14613284

[B24] Costedoat-Chalumeau N. Hulot J.-S. Amoura Z. Delcourt A. Maisonobe T. Dorent R. (2007). Cardiomyopathy related to antimalarial therapy with illustrative case report. Cardiology 107, 73–80. 10.1159/000094079. 16804295

[B25] Cynober L. Aussel C. Vaubourdolle M. Agneray J. Ekindjian O. G. (1987). Modulation of insulin action on 2-deoxyglucose uptake by chloroquine in chick embryo fibroblasts. Diabetes 36, 27–32. 10.2337/diab.36.1.27. 3539675

[B26] Ducharme J. Farinotti R. (1996). Clinical pharmacokinetics and metabolism of chloroquine. Clin. Pharmacokinet. 31, 257–274. 10.2165/00003088-199631040-00003. 8896943

[B27] Duska F. Waldauf P. Halacova M. Zvonicek V. Bala J. Balik M.; Czech Anaesthesia Clinical Trials and Audit Network (2020). Azithromycin added to hydroxychloroquine for patients admitted to intensive care due to coronavirus disease 2019 (COVID-19)-protocol of randomised controlled trial AZIQUINE-ICU. Trials 21, 631. 32641163 10.1186/s13063-020-04566-x PMC7341702

[B28] Erickson T. B. Chai P. R. Boyer E. W. (2020). Chloroquine, hydroxychloroquine and COVID-19. Toxicology Communications 4, 40–42. 10.1080/24734306.2020.1757967. 32457932 PMC7250427

[B29] Estes M. L. Ewing-Wilson D. Chou S. M. Mitsumoto H. Hanson M. Shirey E. (1987). Chloroquine neuromyotoxicity. Clinical and pathologic perspective. Am. J. Med. 82, 447–455. 10.1016/0002-9343(87)90444-x. 3826099

[B30] Fantini J. Di Scala C. Chahinian H. Yahi N. (2020). Structural and molecular modelling studies reveal a new mechanism of action of chloroquine and hydroxychloroquine against SARS-CoV-2 infection. Int. J. Antimicrob. Agents, 55, 105960 10.1016/j.ijantimicag.2020.105960. 32251731 PMC7128678

[B31] Feeney E. Wallace D. Cotter A. Tinago W. Mccarthy C. Keane D. (2020). The COVIRL-001 Trial: a multicentre, prospective, randomised trial comparing standard of care (SOC) alone, SOC plus hydroxychloroquine monotherapy or SOC plus a combination of hydroxychloroquine and azithromycin in the treatment of non- critical, SARS-CoV-2 PCR-positive population not requiring immediate resuscitation or ventilation but who have evidence of clinical decline: a structured summary of a study protocol for a randomised controlled trial. Trials 21, 430 10.1186/s13063-020-04407-x. 32450915 PMC7247433

[B32] Fenollar F. Celard M. Lagier J. C. Lepidi H. Fournier P. E. Raoult D. (2013). Tropheryma whipplei endocarditis. Emerg. Infect. Dis. 19, 1721–1730. 10.3201/eid1911.121356. 24207100 PMC3837638

[B33] Funnell S. G. P. Dowling W. E. Munoz-Fontela C. Gsell P. S. Ingber D. E. Hamilton G. A. (2020). Emerging preclinical evidence does not support broad use of hydroxychloroquine in COVID-19 patients. Nat. Commun. 11, 4253 10.1038/s41467-020-17907-w. 32848158 PMC7450055

[B34] Furst D. E. (1996). Pharmacokinetics of hydroxychloroquine and chloroquine during treatment of rheumatic diseases. Lupus 5 (Suppl. 1), S11–S15. 10.1177/0961203396005001041. 8803904

[B35] Gao J. Tian Z. Yang X. (2020). Breakthrough: chloroquine phosphate has shown apparent efficacy in treatment of COVID-19 associated pneumonia in clinical studies. Biosci. Trends 14, 72–73. 10.5582/bst.2020.01047. 32074550

[B36] Garcia-Cremades M. Solans B. P. Hughes E. Ernest J. P. Wallender E. Aweeka F. (2020). Optimizing hydroxychloroquine dosing for patients with COVID-19: an integrative modeling approach for effective drug repurposing. Clin. Pharmacol. Ther. 108, 253–263. 10.1002/cpt.1856. 32285930 PMC7262072

[B37] Gautret P. Lagier J.-C. Parola P. Hoang V. T. Meddeb L. Mailhe M. (2020a). Hydroxychloroquine and azithromycin as a treatment of COVID-19: results of an open-label non-randomized clinical trial. Int. J. Antimicrob. Agents 56, 105949 10.1016/j.ijantimicag.2020.105949. 32205204 PMC7102549

[B38] Gautret P. Lagier J.-C. Parola P. Hoang V. T. Meddeb L. Sevestre J. (2020b). Clinical and microbiological effect of a combination of hydroxychloroquine and azithromycin in 80 COVID-19 patients with at least a six-day follow up: a pilot observational study. Trav. Med. Infect. Dis. 34, 101663 10.1016/j.tmaid.2020.101663. PMC7151271 32289548

[B39] Gay B. Bernard E. Solignat M. Chazal N. Devaux C. Briant L. (2012). pH-dependent entry of chikungunya virus into *Aedes albopictus* cells. Infect. Genet. Evol. 12, 1275–1281. 10.1016/j.meegid.2012.02.003. 22386853

[B40] Geleris J. Sun Y. Platt J. Zucker J. Baldwin M. Hripcsak G. (2020). Observational study of hydroxychloroquine in hospitalized patients with covid-19. N. Engl. J. Med. 382, 2411–2418. 10.1056/nejmoa2012410. 32379955 PMC7224609

[B41] Gevers S. Kwa M. S. G. Wijnans E. Van Nieuwkoop C. (2020). Safety considerations for chloroquine and hydroxychloroquine in the treatment of COVID-19. Clin. Microbiol. Infect. 26, 1276–1277. 10.1016/j.cmi.2020.05.006. 32422406 PMC7228887

[B42] Goldman F. D. Gilman A. L. Hollenback C. Kato R. M. Premack B. A. Rawlings D. J. (2000). Hydroxychloroquine inhibits calcium signals in T cells: a new mechanism to explain its immunomodulatory properties. Blood 95, 3460–3466. 10.1182/blood.v95.11.3460. 10828029

[B43] Gopel S. Bethge W. Martus P. Kreth F. Iftner T. Joos S. (2020). Test and treat COVID 65 plus - hydroxychloroquine versus placebo in early ambulatory diagnosis and treatment of older patients with COVID19: a structured summary of a study protocol for a randomised controlled trial. Trials 21, 635 10.1186/s13063-020-04556-z. 32650818 PMC7348125

[B44] Guan W.-J. Ni Z.-Y. Hu Y. Liang W.-H. Ou C.-Q. He J.-X. (2020). Characteristics of coronavirus disease 2019 in China. N. Engl. J. Med. 382, 1708–1720. 10.1056/nejmoa2002032. 32109013 PMC7092819

[B45] Hartmann M. Meek I. L. Van Houwelingen G. K. Lambregts H. P. C. M. Toes G. J. Van Der Wal A. C. (2011). Acute left ventricular failure in a patient with hydroxychloroquine-induced cardiomyopathy. Neth. Heart J. 19, 482–485. 10.1007/s12471-011-0185-2. 21826515 PMC3203986

[B46] Hashem A. M. Alghamdi B. S. Algaissi A. A. Alshehri F. S. Bukhari A. Alfaleh M. A. (2020). Therapeutic use of chloroquine and hydroxychloroquine in COVID-19 and other viral infections: a narrative review. Trav. Med. Infect. Dis. 35, 101735 10.1016/j.tmaid.2020.101735. PMC7202851 32387694

[B47] Horby P. Mafham M. Linsell L. L Bell J. Staplin N. Emberson J. R. (2020). Effect of Hydroxychloroquine in Hospitalized Patients with COVID-19: preliminary results from a multi-centre, randomized, controlled trial. medRxiv 10.1101/2020.07.15.20151852.

[B48] Huang C. Wang Y. Li X. Ren L. Zhao J. Hu Y. (2020). Clinical features of patients infected with 2019 novel coronavirus in Wuhan, China. Lancet 395, 497–506. 10.1016/s0140-6736(20)30183-5. 31986264 PMC7159299

[B49] Jeong J.-Y. Choi J. W. Jeon K.-I. Jue D.-M. (2002). Chloroquine decreases cell-surface expression of tumour necrosis factor receptors in human histiocytic U-937 cells. Immunology 105, 83–91. 10.1046/j.0019-2805.2001.01339.x. 11849318 PMC1782639

[B50] Jeong J. Y. Jue D. M. (1997). Chloroquine inhibits processing of tumor necrosis factor in lipopolysaccharide-stimulated RAW 264.7 macrophages. J. Immunol. 158, 4901–4907. 9144507

[B51] Jordan P. Brookes J. G. Nikolic G. Le Couteur D. G. (1999). Hydroxychloroquine overdose: toxicokinetics and management. J. Toxicol. Clin. Toxicol. 37, 861–864. 10.1081/clt-100102466. 10630270

[B52] Joyce E. Fabre A. Mahon N. (2013). Hydroxychloroquine cardiotoxicity presenting as a rapidly evolving biventricular cardiomyopathy: key diagnostic features and literature review. Eur. Heart J. Acute Cardiovascular Care 2, 77–83. 10.1177/2048872612471215. PMC3760572 24062937

[B53] Keyaerts E. Vijgen L. Maes P. Neyts J. Ranst M. V. (2004). *In vitro* inhibition of severe acute respiratory syndrome coronavirus by chloroquine. Biochem. Biophys. Res. Commun. 323, 264–268. 10.1016/j.bbrc.2004.08.085. 15351731 PMC7092815

[B54] Kwiek J. J. Haystead T. A. J. Rudolph J. (2004). Kinetic mechanism of quinone oxidoreductase 2 and its inhibition by the antimalarial quinolines†. Biochemistry 43, 4538–4547. 10.1021/bi035923w. 15078100

[B55] Kwon J.-B. Kleiner A. Ishida K. Godown J. Ciafaloni E. Looney R. J. Jr. (2010). Hydroxychloroquine-induced myopathy. J. Clin. Rheumatol. 16, 28–31. 10.1097/rhu.0b013e3181c47ec8. 20051753

[B56] Kruisselbrink R. J. Zaki Ahmed S. (2010). Acute hydroxychloroquine overdose: case report, literature review, and management recommendations. Am. J. Respir. Crit. Care Med. 181, A6080 10.1164/ajrccm-conference.2010.181.1_meetingabstracts.a6080.

[B57] Kyburz D. Brentano F. Gay S. (2006). Mode of action of hydroxychloroquine in RA-evidence of an inhibitory effect on toll-like receptor signaling. Nat. Rev. Rheumatol. 2, 458–459. 10.1038/ncprheum0292. 16951696

[B58] Löffler B.-M. Bohn E. Hesse B. Kunze H. (1985). Effects of antimalarial drugs on phospholipase A and lysophospholipase activities in plasma membrane, mitochondrial, microsomal and cytosolic subcellular fractions of rat liver. Biochim. Biophys. Acta Lipids Lipid. Metabol. 835, 448–455. 10.1016/0005-2760(85)90114-6. 4016141

[B59] Lagier J.-C. Fenollar F. Lepidi H. Giorgi R. Million M. Raoult D. (2014). Treatment of classic Whipple’s disease: from *in vitro* results to clinical outcome. J. Antimicrob. Chemother. 69, 219–227. 10.1093/jac/dkt310. 23946319

[B60] Le Couteur A. Ung C. Young L. H. Melles R. B. Choi H. K. (2018). Hydroxychloroquine retinopathy - implications of research advances for rheumatology care. Nat. Rev. Rheumatol. 14, 693–703. 10.1038/s41584-018-0111-8. 30401979

[B61] Lee S.-J. Silverman E. Bargman J. M. (2011). The role of antimalarial agents in the treatment of SLE and lupus nephritis. Nat. Rev. Nephrol. 7, 718–729. 10.1038/nrneph.2011.150. 22009248

[B62] Li T. Fan Y. Chen M. Wu X. Zhang L. He T. (2020). Cardiovascular implications of fatal outcomes of patients with coronavirus disease 2019 (COVID-19). JAMA Cardiol. 5, 811–818. 32219356 10.1001/jamacardio.2020.1017 PMC7101506

[B63] Littlejohn E. (2020). Hydroxychloroquine use in the COVID-19 patient. Cleve. Clin. J. Med. [Epub ahead of print] 10.3949/ccjm.87a.ccc011. 32371558

[B64] Liu J. Cao R. Xu M. Wang X. Zhang H. Hu H. (2020). Hydroxychloroquine, a less toxic derivative of chloroquine, is effective in inhibiting SARS-CoV-2 infection *in vitro* . Cell Discov 6, 16 10.1038/s41421-020-0156-0. 32194981 PMC7078228

[B65] Lu R. Zhao X. Li J. Niu P. Yang B. Wu H. (2020). Genomic characterisation and epidemiology of 2019 novel coronavirus: implications for virus origins and receptor binding. Lancet 395, 565–574. 10.1016/s0140-6736(20)30251-8. 32007145 PMC7159086

[B66] Lyngbakken M. N. Berdal J. E. Eskesen A. Kvale D. Olsen I. C. Rangberg A. (2020). Norwegian Coronavirus Disease 2019 (NO COVID-19) Pragmatic Open label Study to assess early use of hydroxychloroquine sulphate in moderately severe hospitalised patients with coronavirus disease 2019: a structured summary of a study protocol for a randomised controlled trial. Trials 21, 485 10.1186/s13063-020-04420-0. 32503662 PMC7273378

[B67] Manku M. S. Horrobin D. F. (1976). Chloroquine, quinine, procaine, quinidine, tricyclic antidepressants, and methylxanthines as prostaglandin agonists and antagonists. Lancet 308, 1115–1117. 10.1016/s0140-6736(76)91090-4. 62951

[B68] Manohar S. Tripathi M. Rawat D. (2014). 4-aminoquinoline based molecular hybrids as antimalarials: an overview. Curr. Top. Med. Chem. 14, 1706–1733. 10.2174/1568026614666140808125728. 25116580

[B69] Manzo C. Gareri P. Castagna A. (2017). Psychomotor agitation following treatment with hydroxychloroquine. Drug Saf Case Rep 4, 6 10.1007/s40800-017-0048-x. 28258476 PMC5336441

[B70] Markus Hoffmann K. M. Hofmann-Winkler H. Kaul A. Kleine-Weber H. Krüger N. Gassen N. C. (2020). Chloroquine does not inhibit infection of human lung cells with SARS-CoV-2. Nature 585, 7826 10.1038/s41586-020-2575-3 32698190

[B71] Marquardt K. Albertson T. E. (2001). Treatment of hydroxychloroquine overdose. Am. J. Emerg. Med. 19, 420–424. 10.1053/ajem.2001.25774. 11555803

[B72] Million M. Lagier J.-C. Gautret P. Colson P. Fournier P.-E. Amrane S. (2020). Early treatment of COVID-19 patients with hydroxychloroquine and azithromycin: a retrospective analysis of 1061 cases in Marseille, France. Trav. Med. Infect. Dis. 35, 101738 10.1016/j.tmaid.2020.101738. PMC7199729 32387409

[B73] Molina J. M. Delaugerre C. Le Goff J. Mela-Lima B. Ponscarme D. Goldwirt L. (2020). No evidence of rapid antiviral clearance or clinical benefit with the combination of hydroxychloroquine and azithromycin in patients with severe COVID-19 infection. Med. Maladies Infect. 50, 384 10.1016/j.medmal.2020.03.006. PMC7195369 32240719

[B74] Muthukrishnan P. Roukoz H. Grafton G. Jessurun J. Colvin-Adams M. (2011). Hydroxychloroquine-induced cardiomyopathy. Circ Heart Fail 4, e7–e8. 10.1161/circheartfailure.110.959916. 21406675

[B75] NIH (2020). NIH halts clinical trial of hydroxychloroquine. [Online] Available at: https://www.nih.gov/news-events/news-releases/nih-halts-clinical-trial-hydroxychloroquine (Accessed August 8, 2020).

[B76] Pastick K. A. Okafor E. C. Wang F. Lofgren S. M. Skipper C. P. Nicol M. R. (2020). Review: hydroxychloroquine and chloroquine for treatment of SARS-CoV-2 (COVID-19). Open Forum Infect. Dis. 7, ofaa130 10.1093/ofid/ofaa130. 32363212 PMC7184359

[B77] Rajasingham R. Bangdiwala A. S. Nicol M. R. Skipper C. P. Pastick K. A. Margaret L. A. (2020). Hydroxychloroquine as pre-exposure prophylaxis for COVID-19 in healthcare workers: a randomized trial. medRxiv 10.1101/2020.09.18.20197327. PMC7665393 33068425

[B78] Randolph V. B. Winkler G. Stollar V. (1990). Acidotropic amines inhibit proteolytic processing of flavivirus prM protein. Virology 174, 450–458. 10.1016/0042-6822(90)90099-d. 2154882

[B79] Raoult D. Drancourt M. Vestris G. (1990). Bactericidal effect of doxycycline associated with lysosomotropic agents on Coxiella burnetii in P388D1 cells. Antimicrob. Agents Chemother. 34, 1512–1514. 10.1128/aac.34.8.1512. 2221859 PMC171863

[B80] Raoult D. Houpikian P. Dupont H. T. Riss J. M. Arditi-Djiane J. Brouqui P. (1999). Treatment of Q Fever endocarditis. Arch. Intern. Med. 159, 167–173. 10.1001/archinte.159.2.167. 9927100

[B81] Recalcati S. (2020). Cutaneous manifestations in COVID‐19: a first perspective. J. Eur. Acad. Dermatol. Venereol. 34, e212–e213. 10.1111/jdv.16387. 32215952

[B82] Richard De-Heer T. D. (2018). A case of hydroxychloroquine induced Hypoglycaemia in a non-diabetic patient. J. Rheum. Dis. and Treat 4, 66 10.23937/2469-5726/1510066.

[B83] Rolain J.-M. Colson P. Raoult D. (2007). Recycling of chloroquine and its hydroxyl analogue to face bacterial, fungal and viral infections in the 21st century. Int. J. Antimicrob. Agents 30, 297–308. 10.1016/j.ijantimicag.2007.05.015. 17629679 PMC7126847

[B84] Rosenberg E. S. Dufort E. M. Udo T. Wilberschied L. A. Kumar J. Tesoriero J. (2020). Association of treatment with hydroxychloroquine or azithromycin with in-hospital mortality in patients with COVID-19 in New York state. JAMA 323, 2493–2502. 10.1001/jama.2020.8630. 32392282 PMC7215635

[B85] Rynes R. I. (1997). Antimalarial drugs in the treatment of rheumatological diseases. Br. J. Rheumatol. 36, 799–805. 10.1093/rheumatology/36.7.799. 9255117

[B86] Salata C. Calistri A. Parolin C. Palu G. (2019). Coronaviruses: a paradigm of new emerging zoonotic diseases. Pathog. Dis. 77, ftaa006 10.1093/femspd/ftaa006. 32065221 PMC7108526

[B87] Sanofi-Aventis (2019). PLAQUENIL®(Hydroxychloroquine sulfate tablets USP). Quebec (Canada): Sanofi-Aventis Canada Inc.

[B88] Savarino A. Boelaert J. R. Cassone A. Majori G. Cauda R. (2003). Effects of chloroquine on viral infections: an old drug against today’s diseases. Lancet Infect. Dis. 3, 722–727. 10.1016/s1473-3099(03)00806-5. 14592603 PMC7128816

[B89] Savarino A. Lucia M. B. Rastrelli E. Rutella S. Golotta C. Morra E. (2004). Anti-HIV effects of chloroquine. J. Acquir. Immune Defic. Syndr. 35, 223–232. 10.1097/00126334-200403010-00002. 15076236

[B90] Schrezenmeier E. Dörner T. (2020). Mechanisms of action of hydroxychloroquine and chloroquine: implications for rheumatology. Nat. Rev. Rheumatol. 16, 155–166. 10.1038/s41584-020-0372-x. 32034323

[B91] Seitz M. Valbracht J. Quach J. Lotz M. (2003). Gold sodium thiomalate and chloroquine inhibit cytokine production in monocytic THP-1 cells through distinct transcriptional and posttranslational mechanisms. J. Clin. Immunol. 23, 477–484. 10.1023/b:joci.0000010424.41475.17. 15031635

[B92] Sheikhbahaie F. Amini M. Gharipour M. Aminoroaya A. Taheri N. (2016). The effect of hydroxychloroquine on glucose control and insulin resistance in the prediabetes condition. Adv. Biomed. Res. 5, 145 10.4103/2277-9175.187401. 27656614 PMC5025914

[B93] Shi S. Qin M. Shen B. Cai Y. Liu T. Yang F. (2020). Association of cardiac injury with mortality in hospitalized patients with COVID-19 in Wuhan, China. JAMA Cardiol 5, 802–810. 10.1001/jamacardio.2020.0950. 32211816 PMC7097841

[B94] Sperber K. Hom C. Chao C. P. Shapiro D. Ash J. (2009). Systematic review of hydroxychloroquine use in pregnant patients with autoimmune diseases. Pediatr Rheumatol Online J 7, 9 10.1186/1546-0096-7-9. 19439078 PMC2690583

[B95] Sperber K. Quraishi H. Kalb T. Panja A. Stecher V. Mayer L. (1993). Selective regulation of cytokine secretion by hydroxychloroquine: inhibition of interleukin 1 alpha (IL-1-alpha) and IL-6 in human monocytes and T cells. J. Rheumatol. 20, 803–808. 8336306

[B96] Stein M. Bell M. J. Ang L. C. (2000). Hydroxychloroquine neuromyotoxicity. J. Rheumatol. 27, 2927–2931. 11128688

[B97] Su S. Wong G. Shi W. Liu J. Lai A. C. K. Zhou J. (2016). Epidemiology, genetic recombination, and pathogenesis of coronaviruses. Trends Microbiol. 24, 490–502. 10.1016/j.tim.2016.03.003. 27012512 PMC7125511

[B98] Tang W. Cao Z. Han M. Wang Z. Chen J. Sun W. (2020). Hydroxychloroquine in patients with mainly mild to moderate coronavirus disease 2019: open label, randomised controlled trial. BMJ 369, m1849 10.1136/bmj.m1849. 32409561 PMC7221473

[B99] U.S. National Library of Medicine (2020a). ClinicalTrials.gov. [Online] Available at: https://clinicaltrials.gov/ct2/results?cond=COVID-19+&term=Hydroxychloroquine+&cntry=&state=&city=&dist= (Accessed July 17, 2020).

[B100] U.S. National Library of Medicine (2020b). Hydroxychloroquine in COVID-19 patients. [Online] Available at: https://clinicaltrials.gov/ct2/show/NCT04394442 (Accessed July 17, 2020).

[B101] U.S. National Library of Medicine (2020c). Hydroxychloroquine in SARS-CoV-2 (COVID-19) Pneumonia Trial. [Online] Available at: https://clinicaltrials.gov/ct2/show/NCT04382625 (Accessed July 17, 2020).

[B102] U.S. National Library of Medicine (2020d). A study of hydroxycholoroquine compared to placebo as treatment for people with COVID-19. [Online] Available at: https://clinicaltrials.gov/ct2/show/NCT04379492 (Accessed).

[B103] Varki A. (1997). Sialic acids as ligands in recognition phenomena. FASEB J. 11, 248–255. 10.1096/fasebj.11.4.9068613. 9068613

[B104] Vincent M. J. Bergeron E. Benjannet S. Erickson B. R. Rollin P. E. Ksiazek T. G. (2005). Chloroquine is a potent inhibitor of SARS coronavirus infection and spread. Virol. J. 2, 1–10. 10.1186/1743-422x-2-69. 16115318 PMC1232869

[B105] Vinciguerra C. Sicurelli F. Fioravanti A. Malandrini A. Battisti C. Federico A. (2015). Hydroxychloroquine neuromyotoxicity: a case with rapid course and complete recovery. Neurol. Sci. 36, 2293–2294. 10.1007/s10072-015-2355-2. 26260758

[B106] Wallace D. J. Gudsoorkar V. S. Weisman M. H. Venuturupalli S. R. (2012). New insights into mechanisms of therapeutic effects of antimalarial agents in SLE. Nat. Rev. Rheumatol. 8, 522–533. 10.1038/nrrheum.2012.106. 22801982

[B107] Wang M. Cao R. Zhang L. Yang X. Liu J. Xu M. (2020). Remdesivir and chloroquine effectively inhibit the recently emerged novel coronavirus (2019-nCoV) *in vitro* . Cell Res. 30, 269–271. 10.1038/s41422-020-0282-0. 32020029 PMC7054408

[B108] Wehbe Z. Hammoud S. Soudani N. Zaraket H. El-Yazbi A. Eid A. H. (2020). Molecular insights into SARS COV-2 interaction with cardiovascular disease: role of RAAS and MAPK signaling. Front. Pharmacol. 11, 836 10.3389/fphar.2020.00836. 32581799 PMC7283382

[B109] WHO (2020a). Coronavirus disease (COVID-19) situation report – 197. [Online] Available at: https://www.who.int/docs/default-source/coronaviruse/situation-reports/20200804-covid-19-sitrep-197.pdf?sfvrsn=94f7a01d_2 (Accessed)

[B110] WHO (2020b). “Solidarity” clinical trial for COVID-19 treatments. [Online] Available at: https://www.who.int/emergencies/diseases/novel-coronavirus-2019/global-research-on-novel-coronavirus-2019-ncov/solidarity-clinical-trial-for-covid-19-treatments(Accessed).

[B111] Wolfe F. Marmor M. F. (2010). Rates and predictors of hydroxychloroquine retinal toxicity in patients with rheumatoid arthritis and systemic lupus erythematosus. Arthritis Care Res. 62, 775–784. 10.1002/acr.20133. 20535788

[B112] Yao X. Ye F. Zhang M. Cui C. Huang B. Niu P. (2020). *In vitro* antiviral activity and projection of optimized dosing design of hydroxychloroquine for the treatment of severe acute respiratory syndrome coronavirus 2 (SARS-CoV-2). Clin. Infect. Dis 71, 732–739. 10.1093/cid/ciaa237. 32150618 PMC7108130

[B113] Zhao H. Wald J. Palmer M. Han Y. (2018). Hydroxychloroquine-induced cardiomyopathy and heart failure in twins. J. Thorac. Dis. 10, E70–E73. 10.21037/jtd.2017.12.66. 29600108 PMC5863196

[B114] Zhou D. Dai S. M. Tong Q. (2020a). COVID-19: a recommendation to examine the effect of hydroxychloroquine in preventing infection and progression. J. Antimicrob. Chemother. 75, 1667–1670. 10.1093/jac/dkaa114. 32196083 PMC7184499

[B115] Zhou P. Yang X.-L. Wang X.-G. Hu B. Zhang L. Zhang W. (2020b). A pneumonia outbreak associated with a new coronavirus of probable bat origin. Nature 579, 270–273. 10.1038/s41586-020-2012-7. 32015507 PMC7095418

[B116] Ziegler H. K. Unanue E. R. (1982). Decrease in macrophage antigen catabolism caused by ammonia and chloroquine is associated with inhibition of antigen presentation to T cells. Proc. Natl. Acad. Sci. Unit. States Am. 79, 175–178. 10.1073/pnas.79.1.175. PMC345685 6798568

